# Risk of Colorectal Cancer in Ulcerative Colitis Patients: A Systematic Review and Meta-Analysis

**DOI:** 10.1155/2019/5363261

**Published:** 2019-11-03

**Authors:** Qing Zhou, Zhao-Feng Shen, Ben-sheng Wu, Cheng-biao Xu, Zhong-qi He, Tuo Chen, Hong-tao Shang, Chao-fan Xie, Si-yi Huang, Yu-gen Chen, Hai-bo Chen, Shu-tang Han

**Affiliations:** ^1^Affiliated Hospital of Nanjing University of Chinese Medicine, Nanjing, Jiangsu, China; ^2^Suzhou Hospital of Traditional Chinese Medicine, Suzhou, Jiangsu, China; ^3^Xuyi Hospital of Traditional Chinese Medicine, Huaian, Jiangsu, China; ^4^Nanjing University of Chinese Medicine, Nanjing, Jiangsu, China; ^5^Shishi General Hospital, Quanzhou, Fujian, China; ^6^The First Affiliated Hospital of Guizhou University of Traditional Chinese Medicine, Guiyang, Guizhou, China

## Abstract

**Background:**

Ulcerative colitis (UC) patients have an increased risk for the development of colorectal cancer (CRC). Our aim was to assess the risk of CRC in UC patients compared with disease extent, disease duration, and geographic variation.

**Methods:**

In this systematic review and meta-analysis, we searched PubMed, scientific meetings, and the bibliographies of identified articles, with English language restrictions for studies published from 1988 to 2018, and assessed the risk of CRC in UC patients. Patients with Crohn's disease, family history of CRC, and colorectal adenomatous polyp (CAP) were excluded from this research. The study was registered with PROSPERO, number CRD42018102213.

**Findings:**

We included 58 studies that included 267566 UC patients. Extensive UC and left-sided UC had a higher risk of CRC than proctitis UC. Geography also played a role in UC-associated CRC development. The time of malignant transformation in Asian UC patients started after 10-20 years of this disease duration. North American UC-associated CRC patients significantly increased in more than 30 years of this disease duration.

**Conclusion:**

In a systematic review of the literature, we found that disease extent, disease duration, and geography were strong, independent risk factors in UC-associated CRC development.

## 1. Introduction

Ulcerative colitis (UC) is an idiopathic, chronic inflammatory disorder of the colonic mucosa, which started in the rectum and generally extended proximally in a continuous manner through part of, or the entire, colon [1]. The clinical course was unpredictable, marked by alternating periods of exacerbation and remission [2]. UC-associated colorectal cancer (CRC) represented a fraction of CRC cases, accounted for up to 5% of all CRC [3]. In contrast to sporadic CRC, UC-associated CRC did not follow the typical “adenoma-carcinoma” sequences [1]. Due to limited understanding of the natural history of UC-associated CRC, the knowledge concerning the CRC risk in UC patients was still inadequate.

The first retrospective analysis on the risk of CRC in UC was published in 1988. In this publication, the overall incidence of CRC in UC was reported as 4.25% [4]. Recent mounting evidences from numerous countries suggested that the CRC standardised incidence ratio (SIR) in UC may differ based on disease duration and geographic variation. For example, the 10-year cumulative probability of cancer after the diagnosis of UC was 4.9%, not having a higher risk of cancer than an age-and sex-matched general population [5]. However, there was no synthesis of risk factors associated with disease extent, disease duration, and geographic variation. Therefore, we did a systematic review and meta-analysis to investigate the risk of CRC in UC patients compared with disease extent, disease duration, and geographic variation.

## 2. Methods

### 2.1. Search Strategy and Selection Criteria

**Figure 1:**
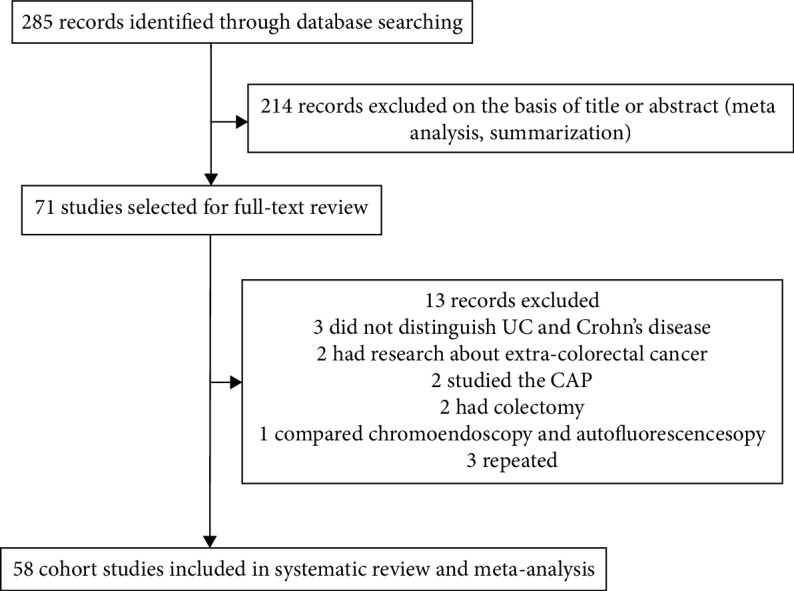
Flow chart of study selection.

We did a comprehensive literature research according to the *Cochrane Handbook for Systematic Reviews of Interventions* and followed the PRISMA and MOOSE guidelines for the reporting of meta-analyses. We searched PubMed, scientific meetings, and the bibliographies of identified articles, with English language restrictions for studies published from 1988 to Dec. 2018. All relevant articles included UC-associated CRC patients. Medical subject heading or keywords used in the search included the following: “Ulcerative Colitis” or “Inflammatory Bowel Disease (IBD)” or “Colorectal Cancer” or “Colorectal Neoplasia”. The full search strategies used for each database were described in Figure 1.

Articles were eligible for inclusion if they reported the UC patients were associated with CRC in terms of the sample size. We included incidence rates in our analyses as an indirect method of adjustment for disease extent, disease duration, and geography.

Two authors (Qing Zhou and Zhao-Feng Shen) independently screened the title and abstract according to these eligibility criteria, screened data extraction, and did quality evaluation. When the evaluation result was not consistent, they consulted other researchers to further resolve differences through consultation according to the literature on the raw data. When the title and abstract met the requirements of the literature, the full text was retrieved for data extraction. NoteExpress 2.0 was adopted to manage the literature, and the repeated literature was removed. The inclusion of the literature was checked according to the abovementioned inclusion criteria, and the related references were traced.

### 2.2. Exclusion Criteria

We excluded the studies in this meta-analysis that met the following inclusion criteria:

1. The literature type did not belong to the category of UC-associated CRC
2. The literature type was a meta-analysis or summarization
3. The literature type data was incomplete (UC and Crohn's disease could not be distinguished effectively) and the additional data could not be further obtained
4. The literature type outcome evaluation index was not CRC
5. The literature type was Crohn's disease, CAP, family history of CRC, or colectomy for UC
6. The literature type was repeated or republished

### 2.3. Data Extraction

Two researchers (Qing Zhou and Zhao-Feng Shen) independently extracted relevant information from all eligible studies using a predefined data extraction form: author, publication year, sample size, age, country, gender, disease extent, and disease duration. Diagnosis and confirmation of UC and CRC were according to the criteria [6]. For missing data, the researchers tried to contact the original literature author by e-mail to obtain relevant data. Data that cannot be obtained was converted according to the relevant requirements of the Cochrane evaluation manual (such as the calculation of standard deviation in continuous data).

### 2.4. Outcomes

The primary outcome measure was the incidence of CRC in UC patients, reported as SIR. We included SIR in our analyses as a direct method of adjustment. No restrictions about publication year, sample size, age, country, gender, disease extent, and disease duration were applied.

The secondary outcomes were measuring the incidence of CRC in UC patients from disease extent, disease duration, geographic variation, and literature reporting time.

### 2.5. Analysis

We used random-effects meta-analysis to assess the incidence of CRC in UC patients. To calculate the pooled SIR of CRC, we combined the extracted study-specific estimates and 95% CIs using the DerSimonian-Laird random-effects model.

Publication bias (small-study effects) was examined with visual assessment of the symmetry of a funnel plot, the asymmetry of which will be assessed through Begg-Mazumdar's rank test. Forest plots were made for the prevalence of the outcomes in overall and within groups.

Data manipulation and statistical analyses were undertaken using the R Software (version 3.4.4). All statistical tests, with the exception of the *Q* statistic, used a two-sided *α* value of 0.05 for significance.

The study was registered with PROSPERO, number CRD42018102213.

### 2.6. Role of the Funding Source

This study was supported by the Ministry of Science and Technology of the People's Republic of China (2017YFC1700602), the National Natural Science Foundation of China (Grant No. 81573978), and the State Clinical Research of TCM (JDZX2015086).

## 3. Results

From 285 articles of potential relevance, 71 full-text articles were examined in detail and 58 studies were included in the final analysis; studies which identified 267566 UC patients, published from Nov. 1988 to Dec. 2018, with 2663 patients that reported UC-associated CRC were included in the meta-analysis. The population characteristics and outcomes of the included studies were summarized in Table 1.

### 3.1. Overall Risk of CRC in UC Patients

**Figure 2:**
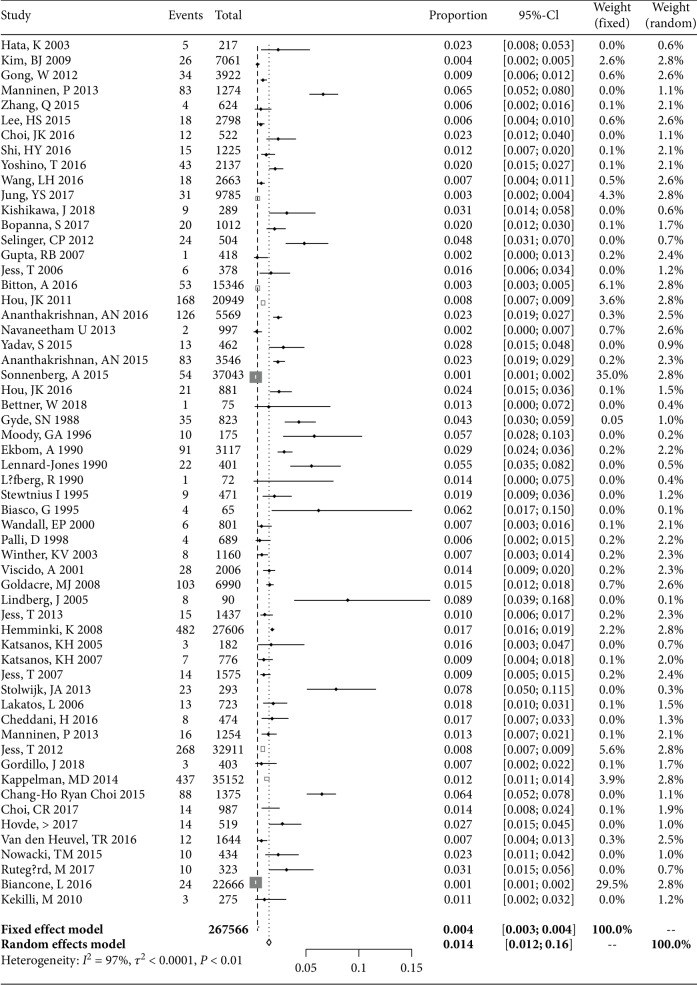
Individual and SIRs of CRC risk in UC: a meta-analysis of population-based cohort studies.

The overall risk of CRC in UC patients among the 58 studies was 1.4% (95% CI: 1.2-1.6; Figure 2). Gender-specific risk estimate for CRC in UC was reported in 30 of the 58 studies and varied from 0.89 (95% CI: 0.56-1.43) to 1.05 (95% CI: 0.68-1.63) in women and men, which has no difference (*P* = 0.62). Disease extent-specific risk estimates for CRC in UC were reported in 21 of the 58 studies, which show that extensive UC and left-sided UC had a higher risk of CRC (SIR: 1.42, 95% CI: 0.83-2.42; SIR: 0.56, 95% CI: 0.38-0.83) than proctitis UC (SIR: 0.18, 95% CI: 0.01-0.03) (*P* < 0.01) (Tables 2 and 3).

### 3.2. Disease Duration Risk of CRC in UC Patients

In the subgroup analysis by disease duration, the incidence of CRC in UC patients rose after 20 years of this disease duration (Table 4).

### 3.3. Geographic Variation Risk of CRC in UC Patients

In the subgroup analysis by geographic variation, Oceania has a higher incidence than other continents; however, it has only one article (Tables 1 and 5). In Europe, the risk of CRC in UC patients has no statistical difference in disease duration for 1-9 years, 10-20 years, 21-30 years, or more than 30 years. In Asia, the risk of CRC in UC increased after 10-20 years of this disease duration. In North America, the risk of CRC in UC increased significantly in more than 30 years of this disease duration (Tables 5 and 6).

Furthermore, we analyzed the CRC incidence in UC patients in each country; we found that Japan, UK, and Austria have the highest incidence, while Canada and Korea have the lowest incidence (Table 7).

### 3.4. The Literature Reporting Time of CRC Risk in UC Patients

In the subgroup analysis by literature reporting time, we found that the risk of CRC in UC patients was higher in 1988-1995. As the research progresses, the CRC risk in UC was stable and maintained between 1.1% and 1.6% (Table 8).

## 4. Discussion

UC, an uncontrolled colorectal inflammation, associated with systemic immune dysregulation, which impaired tumor surveillance, might play a role in colorectal carcinogenesis. Unlike the “adenoma-carcinoma sequence” classically described in sporadic CRC, UC-associated CRC arose from a larger field of colorectal mucosa that was “preconditioned” with a mutational burden that conferred an increased propensity for further dysplasia progression, a phenomenon known as “field cancerization” [55], which followed a sequence of genetic alterations “inflammation-dysplasia-carcinoma” [63]. Chronic colorectal inflammation generated extensive damage to epithelial cells that led to increased cell replication and/or direct DNA damage [1], which was known as one risk factor for the occurrence of CRC in UC patients [28].

This study first provided a picture of the incidence rate of UC-associated CRC from disease extent, disease duration, and geographic variation. Results showed that the overall risk of UC-associated CRC was 1.4%, which increased with disease duration. Extensive UC and left-sided UC had a greater risk of CRC than proctitis UC. There was no obvious gender specificity in CRC risk in UC patients. The strength of this study lies in the fact that we chose to focus on the CRC risk in UC patients from geographic variation. Results showed that the Asian and North American UC patients seemed to have a higher CRC risk. The time of malignant transformation in Asian UC patients started after 10-20 years of this disease duration. North American UC-associated CRC patients significantly increased in more than 30 years of this disease duration.

In this study, we can find that UC patient-relevant endpoint, the risk of CRC, has not decreased over the past decade, and the overall CRC incidence was stable from 1996 until today, which stayed around 1.1% to 1.6%. So the management of UC is still complex, and significant gaps in the literature remained regarding how clinicians could identify the risk associated with CRC and enhance the prevention of UC-associated CRC. 5-Aminosalicylate medications (mesalamine or sulfasalazine), the foundational first-line therapy for the induction and maintenance of mild-to-moderate UC, seemed not to protect against the likelihood of carcinogenesis at doses greater than 2.4 grams daily and still needed to be proven [64]. Other medicines, such as thiopurines, reduced the immunosurveillance of malignant cells and impaired control of oncogenic viruses [65]. Prolonged treatment with thiopurines has been shown to determine an increased risk of a broad range of cancers in UC patients [66]. The impact of UC-related drug therapy on CRC development remained a matter of debate, and the potential benefit of surgery should need to be placed in the context of the risks associated with undertaking complex abdominopelvic reconstructive surgery. Therefore, UC-associated CRC rates still remained a challenging problem for UC patients.

At present, the majority of CRC cases (70%) could be explained by an inadequate surveillance procedure before the CRC diagnosis, and CRC is responsible for approximately 15% of deaths due to UC [67]. Therefore, it is not surprising that researchers have focused efforts on surveillance screening as an adjunct therapy to UC patients for CRC occurrence. UC patients need to be accurately evaluated for the risk of CRC according to the disease extent, disease duration, and geography and need to adhere to a surveillance schedule, such as screening colonoscopy, which should be performed every 1 to 3 years, because the malignant changes and the surrounding inflammation often grow flat and multifocal [68].

## 5. Conclusion

In a systematic review of the literature, we found that the incidence of CRC in UC patients increased with the disease duration. Asian and North American UC patients were more prone to concomitant CRC.

## 6. Limitations

Our study has limitations. Patients with Crohn's disease, CAP, and a family history of CRC and who have undergone colectomy for UC were not included in this study. Patients with UC associated with extracolorectal malignancies (small intestine, blood systems, lung, thyroid, hepatobiliary, skin, melanoma, urinary bladder, breast, genital tract, and so on) were not included, which greatly reduced the risk of cancer in UC. Moreover, our search had English language restrictions. Articles in languages other than English were not included.

## Figures and Tables

**Table 1:** Population and study characteristics.

Author	Sample size (UC)	Study period	Mean age at diagnosis (UC)	Country	Sample size (CRC)	Mean age at diagnosis (CRC)	Gender		Disease extent			Time of duration (year)
							Men	Women	Extensive	Left-sided	Proctitis	
Hata et al. [7]	217	1979-2001	31.4	Japan	5	46.6	0	0	4	1	0	15.2
Kim et al. [8]	7061	1970-2005	37.9	Korea	26	49.6	8	18	0	0	0	12.7
Gong et al. [9]	3922	1998-2009	40	China	34	57.5	14	20	10	12	12	12.8
Matsuoka et al. [10]	1274	1984-2010	30	Japan	83	52	43	40	66	17	10	22
Zhang et al. [11]	624	2000-2012	39	China	4	54.5	2	2	4	0	0	15.5
Lee et al. [12]	2798	1989-2013	33	Korea	18	48	7	11	16	2	0	15
Choi et al. [13]	522	2003-2013	0	Korea	12	0	0	0	0	0	0	0
Shi et al. [14]	1225	1981-2013	41	China	15	0	0	0	0	0	0	0
Yoshino et al. [15]	2137	2003-2013	0	Japan	43	53	28	15	34	8	1	13
Wang et al. [16]	2663	1998-2013	43.1	China	18	0	0	0	0	0	0	19.27
Jung et al. [17]	9785	2011-2014	0	Korea	31	0	20	11	0	0	0	0
Kishikawa et al. [18]	289	1979-2014	33	Japan	9	49	5	4	28	5	0	16
Bopanna et al. [19]	1012	2004-2015	32.1	India	20	53.25	14	6	16	4	0	18.7
Selinger et al. [20]	504	1977-1992	0	Australia	24	0	0	0	12	8	4	0
Gupta et al. [21]	418	1996-1997	26.8	USA	1	0	0	0	0	0	0	0
Jess et al. [22]	378	1940-2001	34	USA	6	50.67	4	2	4	2	0	13.67
Bitton et al. [23]	15346	1999-2008	0	Canada	53	0	37	16	0	0	0	0
Hou et al. [24]	20949	1998-2009	0	USA	168	67	97	71	0	0	0	3.6
Ananthakrishnan et al. [25]	5569	1998-2010	0	USA	126	0	0	0	0	0	0	0
Navaneethan et al. [26]	997	1998-2011	0	USA	2	0	0	0	0	0	0	0
Yadav et al. [5]	462	1940-2011	35	USA	13	66	6	7	0	0	0	31
Ananthakrishnan et al. [27]	3546	2010-2013	0	USA	83	0	0	0	0	0	0	0
Sonnenberg and Genta [28]	37043	2008-2014	49.6	USA	54	0	0	0	0	0	0	0
Hou et al. [29]	881	1999-2014	0	USA	21	0	0	0	0	0	0	0
Bettner et al. [30]	75	1990-2015	29	USA	1	59	0	0	0	0	0	11.1
Gyde et al. [4]	823	1944-1976	33.66	UK/Sweden	35	56.69	13	22	28	7	0	23.03
Moody et al. [31]	175	1972-1981	58.4	UK	10	67	6	4	7	3	0	9.6
Ekbom et al. [32]	3117	1922-1983	0	Sweden	91	0	52	39	35	17	9	0
Lennard-Jones et al. [33]	401	1966-1987	29.73	UK	22	50.05	13	9	0	0	0	20.31
Löfberg et al. [34]	72	1973-1988	0	Sweden	1	42.25	0	0	0	0	0	0
Stewtnius et al. [35]	471	1958-1990	38.3	Sweden	9	49.88	3	6	7	1	1	12.22
Biasco et al. [36]	65	1980-1992	32.9	Italy	4	59.5	0	0	0	0	0	13
Wandall et al. [37]	801	1973-1993	41	Denmark	6	57.33	4	2	4	1	1	10.83
Palli et al. [38]	689	1978-1996	0	Italy	4	0	0	0	0	0	0	0
Winther et al. [39]	1160	1962-1997	0	Denmark	8	0	6	2	0	0	0	0
Viscido et al. [40]	2006	1964-1997	38.5	Italy	28	0	0	0	0	0	0	0
Goldacre et al. [41]	6990	1963-1999	0	UK	103	0	0	0	0	0	0	0
Lindberg et al. [42]	90	1977-2002	0	Sweden	8	0	0	0	0	0	0	0
Jess et al. [43]	1437	1978-2002	0	Denmark	15	0	0	0	0	0	0	0
Hemminki et al. [44]	27606	1964-2004	0	Germany/Sweden	482	0	0	0	0	0	0	0
Katsanos et al. [45]	182	1983-2004	51.2	Greece	3	0	2	1	0	0	0	0
Katsanos et al. [46]	776	1991-2004	0	Netherlands	7	61.3	0	0	0	0	0	5.4
Jess et al. [47]	1575	1962-2005	0	Denmark	14	0	8	6	0	0	0	0
Stolwijk et al. [48]	293	1980-2005	33.8	Netherlands	23	49.2	14	9	22	1	0	10.2
Lakatos et al. [49]	723	2002-2005	36	Hungary	13	51	0	0	8	5	0	19
Cheddani et al. [50]	474	1998-2006	69	France	8	75	0	0	0	0	0	7.4
Manninen et al. [51]	1254	1986-2007	34	Finland	16	47.69	14	2	10	6	0	16.1875
Jess et al. [52]	32911	1979-2008	44.9	Denmark	268	64	132	136	0	0	0	19.1
Gordillo et al. [53]	403	2006-2009	42.91	Spain	3	58.9	3	0	2	1	0	12.5
Kappelman et al. [54]	35152	1978-2010	46.5	Denmark	437	0	0	0	0	0	0	7.8
Choi et al. [55]	1375	2003-2012	30	UK	88	55.5	54	34	0	0	0	25.5
Choi et al. [56]	987	2003-2012	30	UK	14	0	0	0	0	0	0	0
Hovde et al. [57]	519	1990-2013	0	Norway	14	0	10	4	0	0	0	0
Van den Heuvel et al. [58]	1644	1991-2013	45	Netherlands	12	62.75	6	6	4	7	1	3.42
Nowacki et al. [59]	434	2013-2013	45.7	Germany	10	54.7	5	5	6	4	0	12
Rutegård et al. [60]	323	1977-2014	0	Sweden	10	0	0	0	0	0	0	0
Biancone et al. [61]	22666	2012-2014	0	Italy	24	64	0	0	0	0	0	12
Kekilli et al. [62]	275	1994-2008	35.9	Turkey	3	0	0	0	0	0	0	0

**Table 2:** Gender-specific risk of CRC in UC patients.

Gender	Proportion	95% CI
Men	0.0105	0.0068-0.0163
Women	0.0089	0.0056-0.0143

▲*P* < 0.05 vs. women; ▲▲*P* < 0.01 vs. women.

**Table 3:** Disease extent risk of CRC in UC patients.

Extent	Proportion	95% CI
Extensive	0.0142▲▲	0.0083-0.0242
Left-sided	0.0056▲▲	0.0038-0.0083
Proctitis	0.0018	0.0010-0.0031

▲*P* < 0.05 vs. proctitis; ▲▲*P* < 0.01 vs. proctitis.

**Table 4:** Disease duration risk of CRC in UC patients.

Follow-up	Article (*n*)	Proportion	95% CI	Weight (random)
1-9 years	11	0.007	0.005-0.009	22.40%
10-20 years	22	0.013	0.010-0.016	36.60%
21-30 years	11	0.02▲	0.014-0.026	15.60%
More than 30 years	14	0.017▲	0.013-0.022	25.40%

▲*P* < 0.05 vs. 1-9 years; ▲▲*P* < 0.01 vs. 1-9 years.

**Table 5:** Geographic variation risk of CRC in UC patients.

Area	Article (*n*)	Proportion	95% CI	Weight (random)
Asia	13	0.013	0.009-0.017	24.90%
North America	11	0.011	0.007-0.014	22.20%
Europe	33	0.017	0.014-0.020	52.20%
Oceania	1	0.048▲	0.029-0.066	0.70%

▲*P* < 0.05 vs. Europe; ▲▲*P* < 0.01 vs. Europe.

**Table 6:** Geographic variation in UC patients in different disease durations.

Area	1-9 years	10-20 years	21-30 years	More than 30 years
Asia	0.0032 [0.0022; 0.0045] *n* = 1	0.0128 [0.0081; 0.0201]▲ *n* = 6	0.0188 [0.0051; 0.0671]▲ *n* = 4	0.0106 [0.0013; 0.0819]▲ *n* = 2
North America	0.0043 [0.0009; 0.0196] *n* = 4	0.0120 [0.0058; 0.0245] *n* = 5	—	0.0228 [0.0132; 0.0390]▲ *n* = 2
Europe	0.0097 [0.0020; 0.0461] *n* = 6	0.0141 [0.0089; 0.0221] *n* = 10	0.0222 [0.0104; 0.0465] *n* = 7	0.0193 [0.0144; 0.0257] *n* = 10
Oceania	—	0.048 [0.029; 0.066] *n* = 1	—	—

▲*P* < 0.05 vs. 1-9 years; ▲▲*P* < 0.01 vs. 1-9 years.

**Table 7:** CRC incidence in UC patients by country.

Nation	Article (*n*)	Proportion	95% CI	Weight (random)
Canada	1	0.003	0.003-0.004	2.80%
Korea	4	0.005	0.002-0.007	9.30%
Spain	11	0.007	0.000-0.016	1.70%
China	4	0.008	0.006-0.010	9.40%
Italy	4	0.008	0.000-0.016	7.40%
Denmark	6	0.009	0.007-0.012	14.70%
Turkey	1	0.011	0.000-0.023	1.20%
Finland	1	0.013	0.007-0.019	2.10%
USA	10	0.013	0.008-0.017	19.40%
Greece	1	0.016	0.000-0.035	0.70%
France	1	0.017	0.005-0.028	1.30%
Germany	2	0.018	0.016-0.019	3.80%
Hungary	1	0.018	0.008-0.028	1.50%
Netherlands	3	0.019	0.004-0.035	4.80%
India	1	0.02	0.011-0.028	1.70%
Norway	1	0.027	0.013-0.041	1%
Sweden	5	0.027	0.017-0.036	4.50%
Japan	4	0.035	0.012-0.058	4.50%
UK	6	0.039	0.022-0.055	7.50%
Austria	1	0.048	0.029-0.066	0.70%

**Table 8:** The literature reporting time in CRC risk in UC patients.

Reporting time	Article (*n*)	Proportion	95% CI	Weight (random)
1988-1995	6	0.033	0.022-0.043	5.40%
1996-2000	3	0.011	0.000-0.021	4.60%
2001-2005	5	0.014	0.006-0.023	6%
2006-2010	9	0.011	0.005-0.016	6%
2011-2015	17	0.016	0.013-0.020	6%
2016-today	18	0.012	0.009-0.015	6%

## Data Availability

The clinical data supporting this systematic review and meta-analysis are from previously reported studies and datasets, which have been cited. The processed data are available in Table 1 of our manuscript.

## Abbreviations

UC: Ulcerative colitis
CRC: Colorectal cancer
IBD: Inflammatory bowel disease
SIR: Standardised incidence ratio
CAP: Colorectal adenomatous polyp.
